# Food Constituents

**Published:** 1892-12

**Authors:** 


### FOOD CONSTITUENTS.

In looking at the analytical composition of a food we generally find that its constituents are divided into water, ash or mineral matter, albuminoids, nitrogen, free extracts, fats, or oils and fibre. Now to a good many these terms do not convey much idea of the nourishing value of the food. For instance, I was asked the other day why maize containing about 5 per cent. of fat is so much more fattening than linseed cake which contains 10 to 12 per cent. In this case a knowledge of the functions of the different food constituents was wanting, but I think the following will supply all the information necessary to the understanding of a food analysis :—The amount of water in foods varies, and although not different from ordinary water, its natural presence in the food undoubtedly renders the food more palatable, easily digested, and readily assimilated. Ash or mineral matter consists of phosphates of lime, potash, iron, magnesia, and is used to build up the bony framework of the body. Albuminoids, also collectively called protein, form the nitrogenous portions of food. The dried white of an egg is almost pure albumen ; lean meat and casein in milk are good examples, also gluten—the gummy portion—from wheat. The albuminoids of food are used to build muscle (lean meat) and for the casein of milk. For butter production albuminoids serve a most important purpose in food for cows. Probably no mistake is more common in cattle feeding than the giving of too small a quantity of albuminoids to milch cows. Notice the amount of albuminoids in cotton-cake as compared with maize-meal. Nitrogen free extracts consist of sugar, starch, gums, &c., which are carbohydrates. Compare the difference in amount of carbohydrates in maize-meal and cotton-cake. Maize-meal has the greatest quantity of this starchy matter of any food generally used.

The carbohydrates are used by the animal to produce muscular force, heat to keep up the animal warmth, and for the building up of body fat. As regards the fats or oils, all are familiar with the nature of the oils from foods, such as linseed oil and colza oil. They serve about the same purpose in the animal economy that the nitrogen free extract does. One pound of oil will furnish nearly as much heat as 2½ lbs. of starch or carbohydrates. Crude fibre is the woody portion, and of this sawdust, paper, &c., are good examples. The nutritive value of fibre is small, but mechanically it serves a useful purpose in foods for animals that chew the cud or re-masticate their food.

